# Intraoperative Diagnosis of Ochronotic Arthropathy With Unexpected Medial Collateral Ligament Laxity Managed During Total Knee Arthroplasty

**DOI:** 10.7759/cureus.107994

**Published:** 2026-04-29

**Authors:** Saurabh Sah, Prashant Bhavani, Pratik Shahare, Priyanshu, Deepanjan Das

**Affiliations:** 1 Orthopaedics, All India Institute of Medical Sciences, Nagpur, Nagpur, IND

**Keywords:** ligament reconstruction, medial collateral ligament, ochronotic arthropathy, total knee arthroplasty, alkaptonuria

## Abstract

Ochronosis is a rare metabolic disorder caused by alkaptonuria, resulting in the accumulation of homogentisic acid within connective tissues and progressive degeneration of large joints. Clinical presentation often mimics primary osteoarthritis, leading to delayed or missed diagnosis. We report the case of a 54-year-old male who presented with progressive knee pain and was scheduled for total knee arthroplasty for presumed osteoarthritis. No preoperative clinical or radiological features suggested ochronosis. Intraoperatively, extensive black pigmentation of the articular cartilage, menisci, and synovium was observed, raising suspicion of ochronotic arthropathy, which was later confirmed on histopathological examination. During intraoperative gap assessment, unexpected medial collateral ligament laxity was identified, for which no preoperative plan had been made. The instability was successfully managed using available implants, including semitendinosus autograft augmentation secured with titanium interference screws and a cancellous cannulated screw with a washer. Ochronotic arthropathy may present as an unexpected intraoperative finding during total knee arthroplasty, even in the absence of typical preoperative features. Recognition of characteristic intraoperative pigmentation is essential for diagnosis. Medial collateral ligament laxity may be encountered intraoperatively and may require adaptive management. In this case, a satisfactory range of motion and independent ambulation were achieved at follow-up, highlighting the importance of surgical awareness and preparedness. This case highlights that ochronotic arthropathy may present as an intraoperative diagnostic surprise and may be associated with unanticipated ligamentous instability. Awareness of this possibility and readiness to adapt surgical strategy using available resources are essential for achieving optimal outcomes during total knee arthroplasty.

## Introduction

Ochronosis is a rare metabolic disorder resulting from alkaptonuria, an autosomal recessive condition caused by a deficiency of the hepatic enzyme homogentisate 1,2-dioxygenase. This enzymatic defect leads to the accumulation of homogentisic acid, which progressively deposits within connective tissues, resulting in characteristic pigmentation, chronic inflammation, and tissue degeneration [1]. The estimated incidence of alkaptonuria ranges from one in 250,000 to one in one million live births, making ochronosis an infrequently encountered clinical entity [2].

This condition is an extremely rare autosomal recessive metabolic disorder caused by mutations in the homogentisate 1,2-dioxygenase (HGD) gene located on chromosome 3 (region 3q21-q23) [3]. To date, more than 80 different mutations in this gene have been identified in affected individuals. The HGD enzyme, mainly produced in the liver and kidneys, plays a key role in breaking down homogentisic acid (HGA) into maleylacetoacetic acid. When this enzyme is deficient, HGA cannot be properly metabolized and accumulates in the body at levels thousands of times higher than normal [4].

A portion of this excess HGA is eliminated in the urine, which characteristically darkens on exposure to air. Over time, the retained HGA undergoes oxidation and gradually deposits in connective tissues. This process leads to the formation of a dark, melanin-like pigment, a phenomenon known as ochronosis. Clinically, this pigment deposition typically becomes noticeable after the third decade of life and progressively affects cartilage and bone, making them brittle and prone to degeneration. The disease often begins as relatively asymptomatic alkaptonuria but may eventually progress to ochronosis and, ultimately, ochronotic arthropathy, which represents the most severe and disabling manifestation of the condition.

Homogentisic acid deposition most commonly affects articular cartilage, tendons, ligaments, intervertebral discs, sclera, skin, ear cartilage, and cardiac valves [2]. Progressive involvement of large joints leads to cartilage degeneration and secondary osteoarthritis, termed ochronotic arthropathy. The spine, hips, knees, shoulders, and sacroiliac joints are most frequently involved [5]. Clinical symptoms typically manifest after the fourth decade of life and often resemble primary degenerative osteoarthritis, contributing to delayed or missed diagnosis [6].

Diagnosis of ochronotic arthropathy may be established in patients with known alkaptonuria or classic features such as darkened urine or scleral pigmentation. However, in many cases, external manifestations are subtle or overlooked, and the condition may only be recognized incidentally during surgery [7,8]. Intraoperatively, the presence of blackened, brittle cartilage and pigmented synovial tissue is considered pathognomonic [9]. A key distinguishing characteristic of ochronotic cartilage is its increased brittleness, setting it apart from cartilage affected by other types of arthritis. Notably, pigmentation does not involve the bone tissue itself, which remains relatively unaffected by the pathological process [10].

Total knee arthroplasty remains the definitive treatment for advanced ochronotic involvement of the knee [11]. However, surgeons should be aware that ochronosis may also affect periarticular soft tissues, potentially leading to unexpected ligamentous laxity and intraoperative challenges [3]. This case report describes an unexpected intraoperative diagnosis of ochronotic arthropathy during total knee arthroplasty, complicated by unanticipated medial collateral ligament laxity that required immediate intraoperative management.

## Case presentation

A 54-year-old male presented with a four-year history of progressively worsening bilateral knee pain, with the right knee being more symptomatic. Initially, the pain was activity-related and relieved by rest, but over time it became persistent, interfered with daily activities, and failed to respond to conservative treatment, including analgesics and physiotherapy.

The patient denied any history of dark-colored urine, scleral or cutaneous pigmentation, or similar complaints among family members. There was no prior diagnosis of metabolic disease. These features, although often subtle, are recognised clinical indicators of alkaptonuria and may aid in preoperative suspicion when present. On clinical examination, no external signs suggestive of ochronosis were noted. The patient walked with an antalgic gait. Both knees demonstrated neutral alignment without effusion. Tenderness was present along the medial and lateral joint lines. The range of motion of the right knee was limited to zero to 100 degrees, while the left knee demonstrated a range of zero to 120 degrees with pain at terminal flexion.

**Figure 1:**
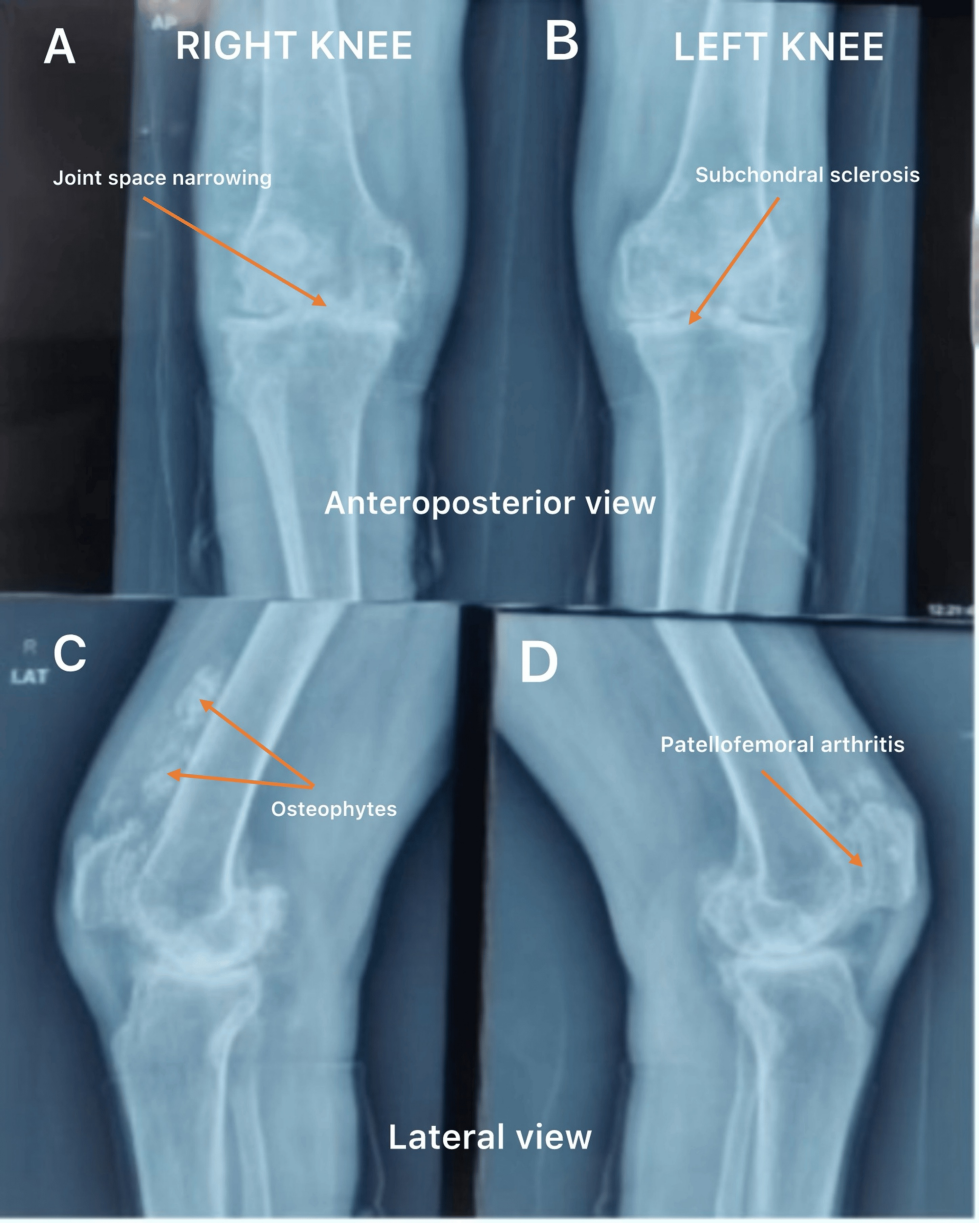
Preoperative radiographs demonstrating degenerative changes in both knees. (A) Anteroposterior view of the right knee showing joint space narrowing.(B) Anteroposterior view of the left knee demonstrating subchondral sclerosis.(C) Lateral view of right knee showing osteophyte formation.(D) Lateral view of left knee demonstrating patellofemoral arthritic changes.

Standing anteroposterior and lateral radiographs of the knees demonstrated concentric joint space narrowing, marginal osteophyte formation, subchondral sclerosis, intra-articular loose bodies, and degenerative changes of the patella, consistent with advanced osteoarthritis. No radiological features specifically suggestive of ochronosis were identified, a finding that has been previously reported in patients with ochronotic arthropathy (Figure 1) [5,7]. The right knee was selected for surgery due to greater symptom severity.

**Figure 2:**
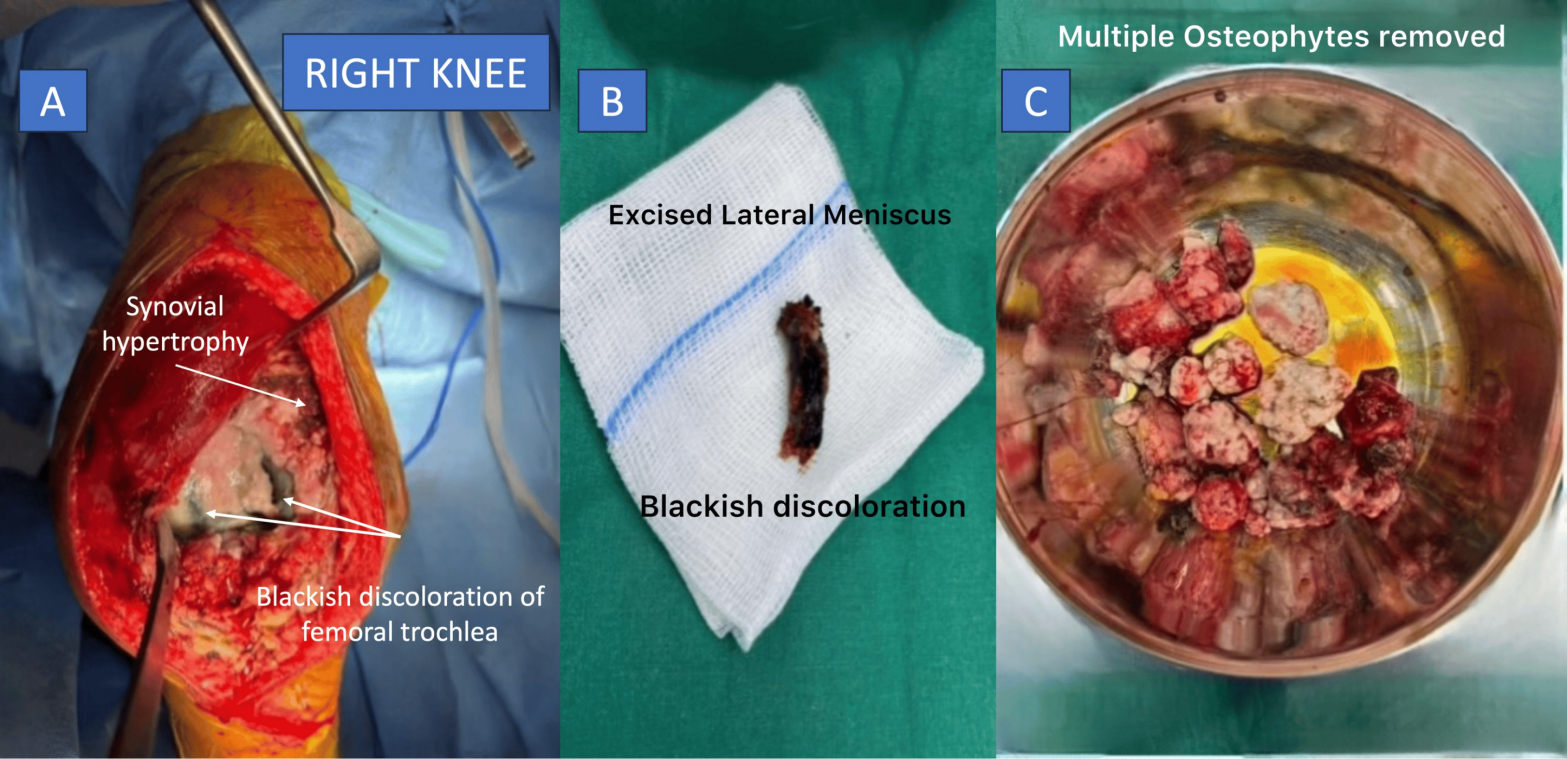
Intraoperative findings in ochronotic arthropathy of the knee. Intraoperative photographs obtained during right total knee arthroplasty demonstrating characteristic features of ochronotic arthropathy.(A) Intraoperative view of the right knee showing marked synovial hypertrophy and blackish discoloration of the femoral trochlea, consistent with ochronotic pigmentation. (B) Excised lateral meniscus exhibiting blackish discoloration. (C)Multiple osteophytes removed during the procedure.

The procedure was performed under spinal anesthesia with epidural analgesia using a standard anterior midline skin incision and subvastus approach. Intraoperatively, marked synovial hypertrophy was observed. The synovium appeared thickened, contracted, and demonstrated striking black pigmentation. Similar black discoloration was noted over the quadriceps tendon and the articular surfaces of the femur, tibia, and patella. The menisci were blackened, brittle, and unusually rigid. Following removal of the cartilage, the underlying subchondral bone appeared macroscopically normal but was subjectively softer than expected, a finding consistent with previously described intraoperative appearances in ochronotic arthropathy (Figure 2) [6,7].

Following distal femoral and proximal tibial bone cuts, assessment of extension and mid-flexion gaps revealed valgus instability due to unexpected laxity of the medial collateral ligament and posterior oblique ligament. As no preoperative ligament reconstruction had been planned, intraoperative decision-making was required. The instability was addressed by augmentation of the medial collateral ligament and posterior oblique ligament using a semitendinosus autograft.

After implantation of the prosthetic components, the semitendinosus tendon was harvested using an open tendon stripper while preserving its tibial insertion. A beath pin was placed at the anatomical femoral footprint of the medial collateral ligament, approximately 3.2 mm proximal and 4.8 mm posterior to the medial epicondyle, and advanced through the lateral femoral cortex under fluoroscopic guidance. The graft was looped around the beath pin, and isometry was confirmed through the full range of motion. A femoral socket measuring 7 mm in diameter and 25 mm in depth was created. With the knee held at thirty degrees of flexion, the graft was secured using a titanium interference screw. The free end of the graft was whip-stitched, passed through a tibial tunnel created five millimeters distal to the joint line on the posteromedial aspect, and fixed on the opposite cortex using a partially threaded cancellous cannulated screw with a washer.

**Figure 3:**
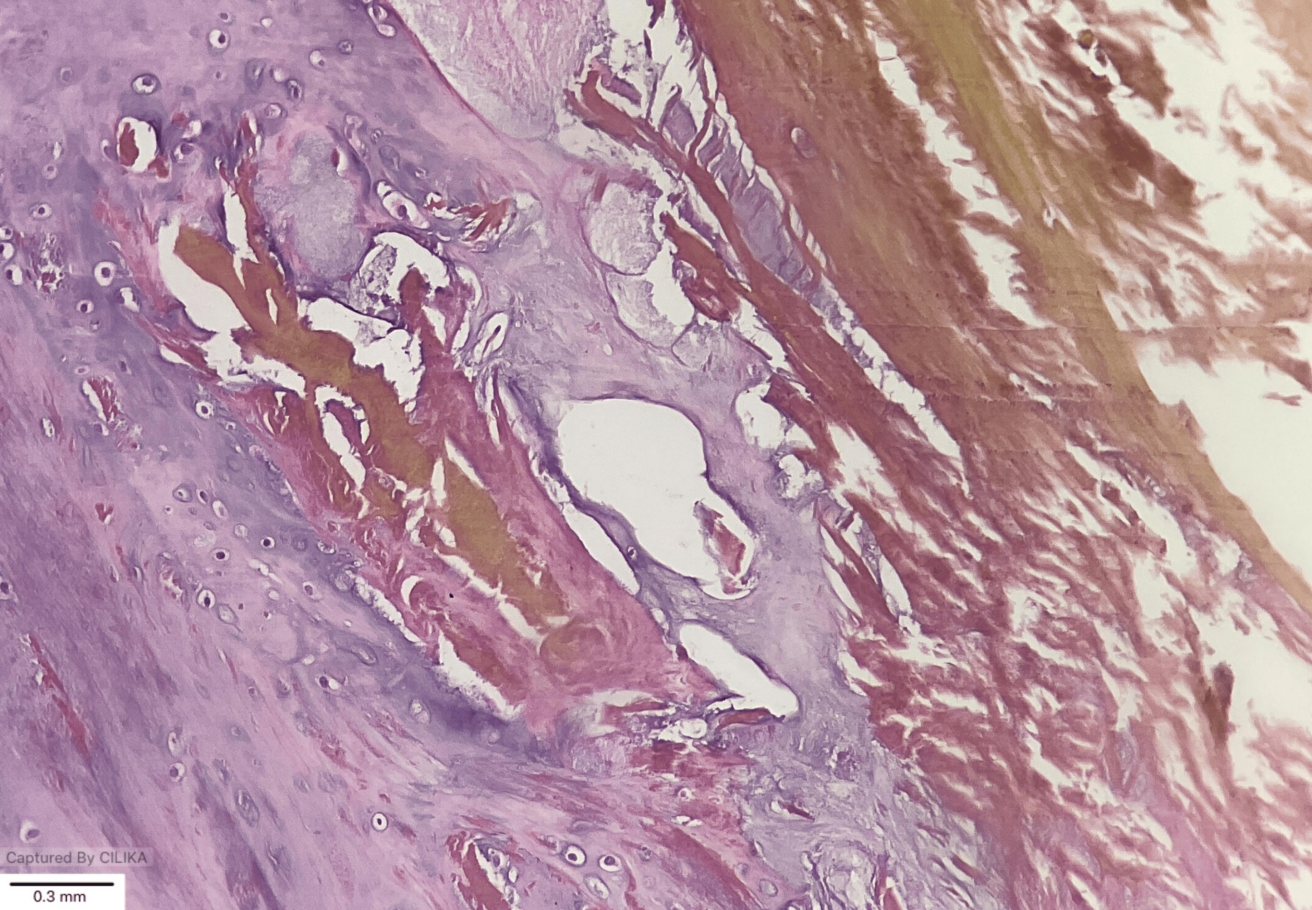
Photomicrograph demonstrating ochronotic pigment deposition within connective tissue, consistent with alkaptonuria. Hematoxylin and Eosin (H&E) and magnification x 20.

The remainder of the procedure was completed without complications. Intraoperative biopsy specimens were sent for histopathological examination, which confirmed the diagnosis of ochronosis (Figure 3).

**Figure 4:**
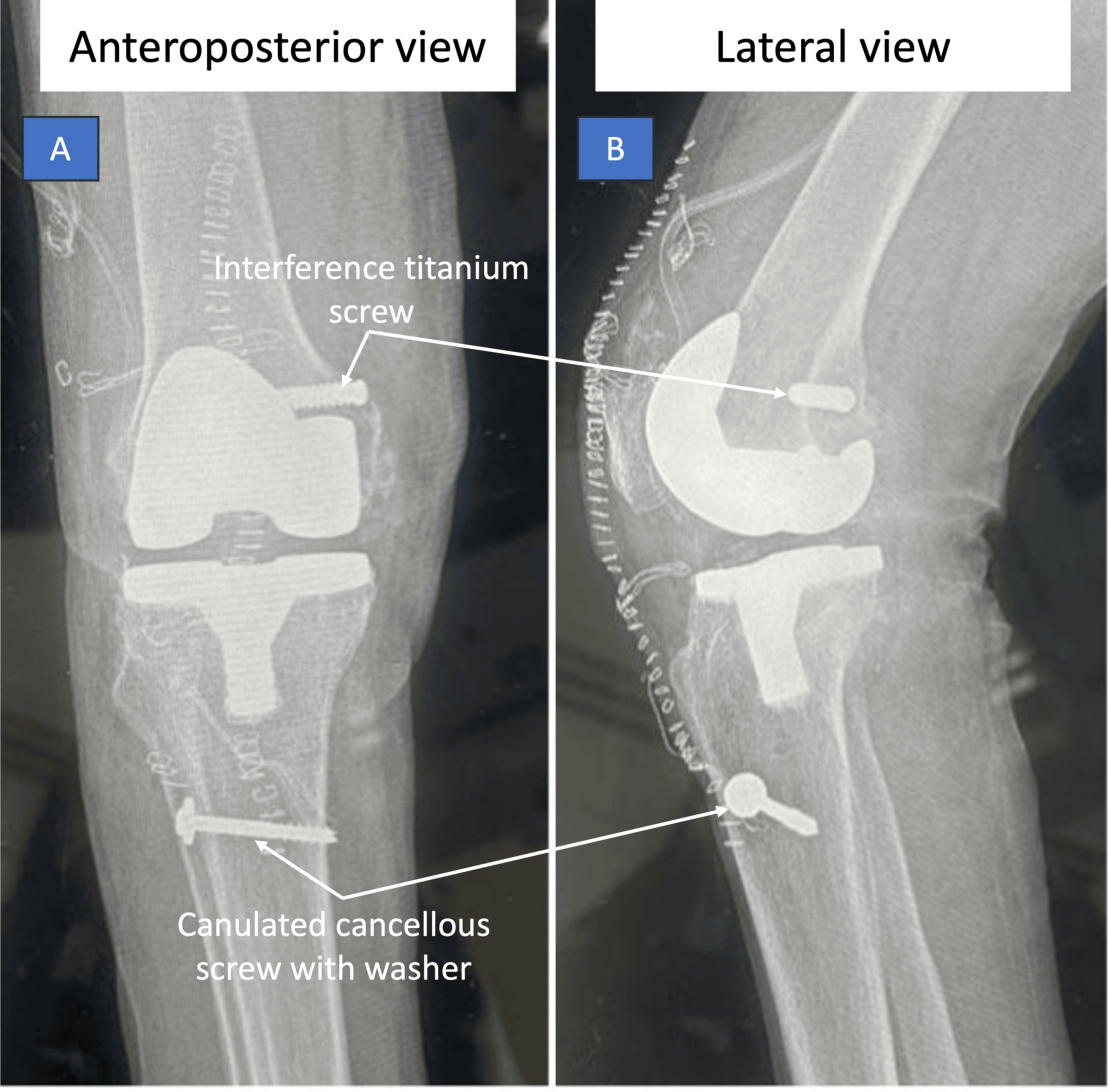
Postoperative radiographs following total knee arthroplasty with ligament reconstruction. (A) Anteroposterior view demonstrating a well-aligned knee prosthesis with a femoral interference titanium screw used for medial collateral ligament fixation. A cannulated cancellous screw with washer is noted on the tibial side securing the posterior oblique ligament graft. (B) Lateral view confirming appropriate positioning of the prosthetic components and fixation hardware, including the femoral interference screw and tibial cannulated cancellous screw with washer.

Postoperatively, anteroposterior and lateral x-rays were done (Figure 4), and the patient was mobilized using a hinged knee brace and maintained non-weight-bearing for six weeks. By the fourth postoperative week, knee flexion of ninety degrees was achieved. Partial weight-bearing was initiated at six weeks. At six months of follow-up, the patient was ambulating independently with full weight-bearing and demonstrated a painless range of motion from zero to 120 degrees (Video 1), findings comparable to outcomes reported in previous arthroplasty series for ochronotic arthropathy [8,9].

**Video 1:** Post operative range of motion of right knee.

The patient subsequently underwent left-sided total knee replacement two months following the right total knee arthroplasty. On follow-up, gait analysis demonstrated a normal, symmetric walking pattern. Additionally, valgus stress testing revealed no medial joint line opening, indicating satisfactory medial stability of the knee (Video 2).

**Video 2:** Post operative valgus stress test of right Knee.

## Discussion

Ochronotic arthropathy is a rare cause of degenerative joint disease resulting from the accumulation of homogentisic acid within connective tissues in patients with alkaptonuria. Clinical manifestations typically appear after the fourth decade of life and often closely resemble primary osteoarthritis, leading to delayed recognition and diagnosis [2,6,12]. As a result, many patients undergo joint arthroplasty without a preoperative diagnosis, with ochronosis being identified only at the time of surgery.

Non-articular signs of alkaptonuria, the underlying metabolic condition, include pigmentation of areas such as the ear lobes, sclera, nose, axillae, and groin. A change in urine color is usually the earliest indicator of alkaptonuria. However, symptoms like urine darkening and pigmentation of the sclera or external ears are frequently unnoticed by patients and their families, which can lead to a delayed diagnosis [6]. In our case, the patient was unaware of any such signs until a clinical examination revealed them.

Intraoperative diagnosis is usually based on the characteristic black or dark brown discoloration of articular cartilage, menisci, and synovial tissue, which is considered pathognomonic [8,9]. Ochronotic arthropathy may be recognized in three ways: in known cases of ochronosis presenting with arthritis, through typical signs like dark urine, tissue pigmentation, and scleral discoloration, or unexpectedly during surgery when characteristic blackened joint tissues are seen in patients without obvious clinical features. In the present case, there were no preoperative clinical, biochemical, or radiological features suggestive of ochronosis, and the diagnosis was established solely based on intraoperative findings and subsequently confirmed by histopathological examination. Similar intraoperative diagnostic surprises have been described in the literature, emphasizing that the absence of classical external manifestations does not exclude the disease [7,9].

Radiographic findings in ochronotic arthropathy may be indistinguishable from primary osteoarthritis, particularly in advanced stages. Although concentric joint space narrowing with relatively minimal osteophyte formation has been described as a typical feature, secondary degenerative changes such as sclerosis and osteophyte formation may be present, as observed in this case [9]. Therefore, radiological evaluation alone is often insufficient to establish a preoperative diagnosis.

In ochronosis, the primary involvement is of the cartilage, while the subchondral bone is generally spared. The patellar tendon may become stiff and fragile due to pigment deposition, so careful handling during patellar retraction is essential. Although the subchondral bone may appear relatively normal, with only mild pigmentation, it can feel unusually soft during bone cuts, sometimes even softer than osteoporotic bone. Hence, extra caution is required during bone preparation to avoid complications. There are reports suggesting patellar tendon avulsion or tear, and also malaligned cuts if these steps are not followed appropriately [13].

Total knee arthroplasty remains the treatment of choice for advanced ochronotic involvement of the knee and has been shown to provide reliable pain relief and functional improvement [1,3,11]. Ochronotic arthropathy is associated with a chronic inflammatory component that can compromise bone quality over time. Therefore, cemented arthroplasty is often preferred, as it may provide more reliable fixation and help reduce the risk of implant subsidence and loosening in the long term [14]. However, most reports primarily focus on osseous and cartilaginous involvement, with limited discussion regarding the condition of periarticular soft tissues. In the present case, unexpected medial collateral ligament and posterior oblique ligament laxity was identified during intraoperative gap assessment, despite no preoperative clinical evidence of instability.

Ligamentous involvement in ochronosis is likely related to homogentisic acid deposition within collagen-rich tissues, leading to structural weakening and loss of elasticity. Although rarely reported, such soft tissue compromise may result in intraoperative instability and present a significant surgical challenge [3]. In this case, no preoperative plan had been made for ligament reconstruction, necessitating real-time intraoperative decision-making. Stability was successfully achieved using a semitendinosus autograft secured with readily available implants, including a titanium interference screw and a cancellous cannulated screw with a washer.

In ochronotic arthropathy, direct comparison of arthroplasty outcomes with those seen in other metabolic, degenerative, or inflammatory disorders is difficult due to its unique disease characteristics and progression [15]. This case underscores the importance of surgeon awareness and adaptability when encountering unexpected intraoperative findings during routine arthroplasty. Preparedness to recognize ochronosis and manage associated ligamentous instability using available resources is essential to ensure optimal outcomes. Despite the metabolic nature of the disease and unanticipated soft tissue compromise, total knee arthroplasty with appropriate intraoperative ligament reconstruction can yield excellent functional results.

## Conclusions

Ochronotic arthropathy may present as an unexpected intraoperative finding during total knee arthroplasty, even in the absence of typical preoperative features. This case illustrates that associated ligamentous laxity can occur and may require intraoperative adaptability. Awareness of this possibility can aid surgical decision-making and help achieve satisfactory outcomes in similar scenarios.
